# Coping, acculturation, and psychological adaptation among migrants: a theoretical and empirical review and synthesis of the literature

**DOI:** 10.1080/21642850.2013.843459

**Published:** 2014-01-02

**Authors:** Ben C.H. Kuo

**Affiliations:** ^a^ Department of Psychology, University of Windsor, 401 Sunset Ave., Chrysler Hall South, Windsor, Ontario, CanadaN9B 3P4

**Keywords:** coping, stress, acculturation, cultural, migrant

## Abstract

Given the continuous, dynamic demographic changes internationally due to intensive worldwide migration and globalization, the need to more fully understand how migrants adapt and cope with acculturation experiences in their new host cultural environment is imperative and timely. However, a comprehensive review of what we currently know about the relationship between coping behavior and acculturation experience for individuals undergoing cultural changes has not yet been undertaken. Hence, the current article aims to compile, review, and examine cumulative cross-cultural psychological research that sheds light on the relationships among coping, acculturation, and psychological and mental health outcomes for migrants. To this end, this present article reviews prevailing literature pertaining to: (a) the stress and coping conceptual perspective of acculturation; (b) four theoretical models of coping, acculturation and cultural adaptation; (c) differential coping pattern among diverse acculturating migrant groups; and (d) the relationship between coping variabilities and acculturation levels among migrants. In terms of theoretical understanding, this review points to the relative strengths and limitations associated with each of the four theoretical models on coping-acculturation-adaptation. These theories and the empirical studies reviewed in this article further highlight the central role of coping behaviors/strategies in the acculturation process and outcome for migrants and ethnic populations, both conceptually and functionally. Moreover, the review shows that across studies culturally preferred coping patterns exist among acculturating migrants and migrant groups and vary with migrants' acculturation levels. Implications and limitations of the existing literature for coping, acculturation, and psychological adaptation research are discussed and recommendations for future research are put forth.

## Introduction

Globalization and migration mark the rhythm and the tempo of the contemporary modern society. On-going movement and mobility of people across continental, national, and regional boundaries are now an everyday experience and norm for many. The demographic characteristics of present-day migrants internationally speak to this reality clearly. For instance, International Organization for Migration (2013) documented that an estimate of 214 million individuals worldwide are considered to be international migrants in 2013. This denotes a dramatic jump of more than 40% from 150 million just over a decade ago in 2000. This number stands for 3.1% of world's population, suggesting one in every 33 persons in the world's population is a migrant (International Organization for Migration, 2013). This extraordinary, continuous expansion of movement of people globally has brought about many exciting opportunities as well as unprecedented new challenges.

For the last three decades within the discipline of psychology, the research on acculturation stands as a major force of scientific inquiry that concerns directly with migration and cultural change. Specifically, acculturation research represents an overriding focus of scholarly work within cross-cultural and multicultural psychology (Sam & Berry, 2006). Despite some recent criticisms (Rudmin, 2003), acculturation theory embodies an important empirically driven and practically useful framework to help discern cultural transition for migrants moving from one cultural context/environment to another in terms of the elements, the processes, and the consequences of migration and cultural transition. Incidentally, prominent acculturation scholars (Berry, 1997, 2006; Ward, 2001) have asserted that the principles of acculturation theory are deeply grounded in the broader psychological theory of stress and coping (Lazarus & Folkman, 1984). From this theoretical vintage point, acculturation or cultural adaptation is viewed as an inevitable process human species undergo in an effort to manage and cope with stressors and changes brought upon by migration and by being in a prolonged contact with a new, host culture (Berry, 1997). Consequently, there exists an interwoven relationship between coping and acculturation for individuals undergoing cultural change and transition.

While there have been frequent discussions alluding to the coping-acculturation association in the existing acculturation literature, surprisingly systematic scholarly efforts to discern and integrate current knowledge on coping and acculturation for migrant populations cannot be located by this author. This observation is further underscored by the fact that scholars in this area have noted the scarcity of current research examining coping's role in cross-cultural transition generally (Ward & Kennedy, 2001) and in the lives of recent immigrants specifically (Yakushko, 2010; Yakushko, Watson, & Thompson, 2008).

Cast within this above context, this present article intends to survey and review prevailing psychological theories and empirical research pertaining to coping, acculturation, and psychological adaptation among migrants. In this paper, the term ‘migrant’ is being used inclusively, to refer to individuals who engage in *voluntary* and *involuntary* migration. This term includes immigrants, refugees, international students/sojourners, migrant works and members of ethnic minority groups (i.e. immigrants of later generations) (Bemak & Chung, 2008). In keeping with the objectives of this special issue of *Health psychology and behavioral medicine* on ‘Migration and Health’, this review focuses specifically on the psychological and the mental health aspects of adaptation for migrants; that is, in terms of general stress, acculturative stress, perceived discrimination, etc. This article asks and attempts to address two critical questions: ‘What do we currently know about coping's role and effect on the process of acculturation for and the psychological/emotional/mental health well-being of migrants?’ and, more specifically ‘How does acculturation affect migrants' coping behaviors and preferences in dealing with general and acculturative stress?’. To address these questions, this article will review, summarize and evaluate current understanding and research in terms of: (a) conceptual perspectives on coping, acculturation, and psychological/mental health outcomes; (b) four theoretical models of stress, coping, and acculturation/cultural adaptation; (c) differential coping patterns among diverse acculturating groups of migrants and ethnic populations; and (d) the relationship between coping variabilities and acculturation levels for migrants and ethnic populations. At its conclusion, this review will also discuss and offer recommendations for future research in the area of coping and acculturation for migrants.

### Definitional and contextual issues of coping and acculturation

Research on stress and coping represents a mainstay of psychological inquiry as evidenced by the proliferation of published stress–coping studies stemming from social, clinical, and health psychology research over the last three decades (Aldwin, 2007; Folkman & Moskowitz, 2004). The classic definition of coping by Lazarus and Folkman (1984) describes coping as: ‘the constantly changing cognitive & behavioral efforts to manage specific external and/or internal demands that are appraised as taxing or exceeding the resources of the person.’ (p. 141). The exceptional popularity of studying individuals' coping within psychological research can be attributed to: (a) the critical role coping plays as a mediator between the stressor or adversity and the health consequences for an individual; (b) the resultant effects coping have on the physical or psychological outcomes of an individual; (c) the complex and intricate relationship between coping and multiple psychosocial protective factors, such as social support, resilience, positive affect, and subjective well-being; and (d) the clinical implications of enhancing individuals' adaptive coping behaviors in promoting well-being (Aldwin, 2007; Kuo, Arnold, & Rodriguez-Rubio, 2013).

While psychological research on coping and its efficacy abounds, there has been comparatively less attention and effort made to elucidate culture and its influences on the responses and the processes of coping. As a case in point, Heppner (2008) noted that US coping and applied problem-solving scholars have studied the subject of coping in a ‘culture-blind’ manner that largely overlooked the cultural context in which stress and coping occur. In response to such a criticism, there have been increasing calls and attempts, mainly by cross-cultural and cultural coping researchers, to consider culture's role and effect on the stress and coping process (Aldwin, 2007; Chun, Moos, & Cronkite, 2006; Wong & Wong, 2006, for examples). These scholarly discussions and analyses have begun to shed lights on how and why cultural differences and specificities in coping patterns and preferences exist among culturally diverse populations, including migrants across ethnic and racial lines (Kuo, 2011, 2013).

Parenthetically one way in which stress responses and coping behaviors have been conceptually and empirically linked to cultural context and influence is observed in the limited, but growing corpus of research on coping with acculturation and/or acculturative stress for individuals undergoing cultural transition (Kuo & Roysircar, 2004; Noh & Kaspar, 2003; Torres & Rollock, 2004; Yakushko, 2010). In parallel to stress and coping research, acculturation research has assumed a prominent role in cross-cultural and multicultural psychological research for the past three decades (Yoon et al., 2012). Redfield, Linton, and Herskovits (1936) defined acculturation as a phenomenon that occurs: ‘when groups of individuals having different cultures come into continuous first-hand contact with subsequent changes in the original culture patterns of either or both groups’ (p. 149). As such, the advancement in acculturation research in recent time corresponds to the increasing transcontinental migration of people worldwide due to economic globalization and intergroup conflicts. This is underscored by a rise in empirical studies on the acculturation and cultural adaptation experiences of diverse groups of immigrants and refugees (Yakushko et al., 2008), international students (Kuo & Roysircar, 2006; Popadiuk, 2009) and racial and ethnic minorities (Castro & Murray, 2010).

Acculturation has a strong conceptual and empirical appeal in sociological and psychological research because of its hypothesized as well as demonstrated relationships to a wide array of psychosocial factors among migrants, including mental health (Yoon et al., 2012), sociocultural adaptation (Ward & Kennedy, 2001), acculturative stress (Kuo & Roysircar, 2004; Torres & Rollock, 2004), self identity and personality (Ryder, Alden, & Paulhus, 2000), family relationship quality (Hwang, 2006), to name a few. Lying at the core of acculturation theory is the person-environment transactional theoretical paradigm of stress and coping as propositioned by Lazarus and Folkman (1984) (Berry, 1997; Ward, 2001). The conceptual significance of ‘acculturative stress’ within the acculturation theory further attests to the theory's lineage to the stress–coping paradigm (Berry, 1997, 2006). In essence, stress and coping are conceptualized as inherent and inevitable aspects of acculturating experiences and processes for individuals undergoing the migration process. The following sections aim to take stock of and illustrate what we currently know about the interconnections among stress, coping, acculturation, and psychological/mental health adaptation for migrant and ethnic populations.

### Conceptualization of acculturation and adaptation from a stress and coping perspective

As mentioned previously, the conceptual principles of stress and coping theory undergird the foundation of contemporary acculturation theory and research (Berry, 1997; Cervantes & Castro, 1985). Berry (2006) noted that cultural adaptation of migrants can be hypothesized through two distinct perspectives: the ‘cultural learning/shedding’ model vs. the ‘stress, coping, and adaptation’ model. The cultural learning model supposes that cultural adaptation occurs through migrants' learning of culture-specific skills that would enable them to negotiate their ways in the new cultural environment (Ward, 2001). Under this framework, the emphasis is placed on teaching and training migrants or newcomers new knowledge, new language, and new intercultural skills. In contrast, the stress, coping, and cultural adaptation model has been argued to be a more flexible explanatory framework to discern acculturation because it can address acculturating individuals' responses to conflicts and stresses arising from intercultural contacts. Explicitly this latter perspective ‘incorporates both characteristics of the individual and characteristics of the situation that may facilitate or impede adjustment to a new cultural milieu’ (p. 413; Ward, 2001). In fact the stress and coping perspective stands as the most popular and widely applied conceptual scheme in studying acculturation and cultural transition in the existing literature (Ward & Kennedy, 2001) and is expected to continue to dominate acculturation research for many years to come (Ward, 2001).

According to Lazarus and Folkman's (1984) seminal work, the experience of stress and coping becomes most salient when individuals are faced with major life changes or challenges. Immigration or migration constitutes an example of such a major life event for migrants. Expectedly coping responses to counter stresses resulting from cultural transition are natural and inevitable aspects of acculturation and cultural change for most immigrants, refugees, international students or sojourners, and even for ethnic individuals of immigrant parents. In this regard, the existing acculturation literature has identified a constellation of acculturation-related stressors frequently faced by migrants that demand coping responses (Bemak & Chung, 2008; Castro & Murray, 2010; Kuo & Roysircar, 2006). In a recent literature review, Yakushko et al. (2008) summarized stressors commonly faced by immigrants and refugees. They include: (a) pre-migration stressors, such as previous experiences with violence, conflict, and trauma, and fear associated with migrants' flight; and (b) post-migration stressors related to relocation, mental and physical health problems, acculturative stress, loss of social status and contact, and oppression by the host society. Moreover, Lazarus and Folkman (1984) assert that different individuals facing a stressor of similar nature can have divergent experiences and physical and psychological reactions, due to individuals' predispositions in: (a) primary appraisal – the extent to which a stressor is viewed as a threat; and (b) secondary appraisal – the evaluation and assessment of one's resources to respond to the stressor. This second level of appraisal speaks directly to coping strategies that would most likely be evoked in dealing with the target stressor by the individual and would subsequently impact the person's well-being and adaptation.

Following this hypothesized stress–coping–acculturation relationship, it is not unexpected that coping plays a central role in major contemporary theories of acculturation and cultural adaptation. As explicated by Berry (2006), the terms ‘stress’ and ‘coping’ bear significant theoretical implication and meaning in discerning how migrants deal with negative experiences arising from intercultural contacts. People are viewed as having the potential to cope with stressors to achieve ‘a variety of outcomes (adaptations), ranging from very negative through very positive’ (p. 43, Berry, 2006). To further explore and elucidate the above described relationships, this article will now turn to consider the hypothesized links among stress, coping, acculturation, and adaptation of migrants from four existing theories.

### Theories of coping, acculturation, and adaptation

A careful search of the existing psychological research by the author reveals that there are only a handful of comprehensively articulated conceptual models of acculturation or cultural adaptation currently in the published psychological literature. Specifically four of these theoretical models were identified to have specifically implicated and hypothesized coping behaviors in the process and the outcome of migrants' long-term acculturation and adaptation. Given the specific objective of the current paper on coping and acculturation, these theoretical models will be described and reviewed with a specific focus on the hypothesized role and the function of coping in relation to migrants' acculturation and adaptation. For a fuller and more comprehensive discussion on each of these theories, readers are encouraged to refer to the original publications of these theories. These acculturation theories are presented chronologically in the order in which the theories were published.

#### Multivariate stress–mediation–outcome model for Mexican Americans by Cervantes and Castro (1985)

Richard Cervantes and Felipe Castro's (1985) stress–mediation–outcome theory is among the earliest conceptual model of cultural adaptation in the literature. It addresses the relationships among stress, stress responses, and mental health consequences for immigrants. The model was established based on an extensive review of psychology literature on Mexican Americans. As Cervantes and Castro noted, their conceptual attempt was intended to capture and examine factors of ‘clinical importance in prompting a better understanding of the experience of stress and its relationship to various forms of psychopathology’ (p. 2) for Mexican Americans. Stemming from this objective, this model highlights the impact of the stress–coping–adaptation for Mexican Americans in terms of ‘mental health’ outcomes and implications for clinical interventions.

This multivariate model conceptualizes cultural adaptation of Mexican American immigrants and their children as an interplay among five ‘generic factors’: (a) potential stressors; (b) appraisal; (c) external mediators; (d) internal mediators; and (e) coping responses. Under the first factor, potential stressors, Cervantes and Castro (1985) contended that common stresses experienced by Mexican American immigrants can include stresses associated with the demand of cultural changes, loss of status for immigrants, and/or economic difficulties such as poverty. Appraisal entails a cognitive evaluation of the extent of the stressor in question. These authors postulated that this stress appraisal interacts with and is affected by a whole host of external factors (e.g. family support, non-family support, and mental health services) and internal factors (e.g. personality traits, gender role, etc.) associated with an immigrant. Finally, coping responses are believed to act as a secondary mediator between appraisal, internal factors, and external factors on the one hand, and short- and long-term mental health and adaptation consequences (e.g. depression, psychological distress, and substance abuse) on the other for Mexican American immigrants.

On the basis of their proposed multivariate stress–mediation–outcome model, Cervantes and Castro (1985) advocated cross-cultural research to incorporate all of the hypothesized variables and to test the pathways among them in acculturation research with Mexican American populations. They further stressed the need to identify and study culture-specific stress appraisal processes and coping patterns among subgroups of Mexican Americans during the process of acculturation and cultural adjustment. They also urged cross-cultural research to investigate the appraisal and the coping processes of Mexican Americans in comparison with other ethnic groups in the USA. Even though Cervantes and Castro's (1985) Multivariate stress–mediation–outcome theory of cultural adaptation has been published for nearly two decades, it is not clear if or to what extent this model has been applied to empirically study coping and acculturation for Mexican Americans or any other cultural groups. However, a recently modified and expanded version of Cervantes and Castro's theory is found in a newer theoretical model proposed by Castro and Murray (2010) and it will be reviewed latter in this section

#### Acculturation strategies framework by Berry (1997)

Berry's (1997) formulation of cultural adaptation represents the pioneering and the most influential work on acculturation. Berry's (1997) seminal theory of acculturation is grounded in Lazarus and Folkman's (1984) early work on stress and coping. At one level, the four ‘acculturation strategies’, *assimilation*,* separation*,* marginalization*, and *integration*, adopted by acculturating groups or individuals is said to embody the coping attempts they adopt to manage their relationship with the host/dominant cultural group (Berry, 1997, 2006). The theory further distinguishes acculturation in terms of ‘group-level’ and ‘individual-level’ impacts. In the model, specific group-level factors and individual-level factors occur prior to and during acculturation can act on the process and the outcome of migrants' adaptation. At the group level, on the other hand, coping behaviors are manifested through the following process. Acculturation group undergoing cultural transition first appraise the adjustment-related stressors then come up with coping strategies to deal with these stressors. The selected coping behaviors will act on the stressor and produce certain effects. This will subsequently lead to long-term outcomes for the group, ideally in a successful adaptation.

At the individual or psychological level, the theory points to a set of moderating variables for a person *prior to* acculturation; these encompass an individual's age, gender, education, pre-acculturation, status, migration motivation, expectations, cultural distance (language, religion, etc.), and personality characteristics (locus of control, flexibility). In addition, individual-level moderating variables occurring *during* acculturation can include phase of acculturation (length of time), acculturation strategies (attitudes and behaviors), coping strategies and resources, social support, and social attitudes (prejudice and discrimination) (Berry, 1997). It is at this juncture of the acculturation process Berry' model stresses the particular importance of coping. Coping is necessary for migrants to deal with psychological difficulties during acculturation and can lead to migrants' eventual adaptation in the new culture (Berry, 2006).

According to Berry's theory (1997, 2006), the first type of adaptation involves ‘behavioral shift’ on the part of the acculturating individual, to adopt the ways of the dominant culture in order to reduce stress or intergroup conflict. The second type pertains to ‘psychopathology’ which typically refers to unsuccessful coping and acculturation experience on the part of the individual and it can lead to high stress, such as in the form of separation (withdrawal) or marginalization. The third and final type of adjustment involves ‘acculturative stress’ which refers to a source of stress commonly faced by immigrants, refugees and indigenous people when coming in contact with members of the outside, dominant cultural group (Berry, Kim, Minde, & Mok, 1987). Acculturative stress is characterized by negative behavioral and emotional reactions attributable to the process and experience of adjusting to a new cultural milieu (Berry et al., 1987). Berry (2006) observed that to respond to acculturative stress, individuals use a wide range of coping methods from adaptive to non-adaptive strategies, and these coping variabilities can lead to varying levels of acculturative stress experienced by migrants.

In short, Berry's acculturation strategies theory stipulates that the quality of acculturation for a migrant in the host/dominant cultural environment is contingent upon his/her ability to cope appropriately and effectively with stresses associated with cultural adaptation. Berry called for further research to expound on the relationships between the four acculturation strategies, *assimilation*,* separation*,* marginalization*, and *integration*, and the three major types of coping, *problem-focused*,* emotional-focused*, and *avoidance coping*. Without a doubt Berry's acculturation strategies theory embodies the cornerstone of acculturation research and is widely cited and researched both within the field of psychology and beyond (Sam & Berry, 2006). As such, in comparison with the other three acculturation and cultural adaptation theories reviewed in this section, Berry acculturation strategies theory is clearly the most extensively applied and empirically investigated acculturation model.

#### Resilience-based stress–appraisal–coping model by Castro and Murray (2010)

Building on and expanding upon Cervantes and Castro's (1985) original stress–mediation–coping model discussed earlier, Castro and Murray (2010) proposed a newer and more refined developmental model of stress, coping, and acculturation/cultural adaptation. Unlike its earlier predecessor, Castro and Murray's (2010) stress–appraisal–coping scheme is grounded in the theory and research of resilience and it conceptualizes coping and cultural adjustment within a longitudinal, temporal, and developmental framework. Within this perspective ‘resilience’ or ‘positive cross-cultural adaptation’ is defined as the outcome of adaptation resulting from migrants' ‘persistent efforts at coping with multiple and often chronic stressors encountered within the new environment’ (p. 376; Castro & Murray, 2010). Cultural adaptation process is further stipulated as the ‘resilience trajectory’ – a pathway through which migrants undergo in their host society over time. Hence, coping abilities and resources on the part of migrants are viewed as essential in fostering and facilitating their resilient psychosocial adjustment. Hence coping plays a central role in this resilience-based stress–appraisal–coping conceptual framework.

In particular, this developmental model highlights the following aspects of coping and cultural adaptation. First, Castro and Murray (2010) hypothesize that migrants' resilience in cultural adaptation is a product of the interactions among multiple individual, familial, and community factors. This is because these factors bear dynamic influences on migrants' process of settlement as they enter into a new cultural context. Second, the model submits that the adaptation pathway of migrant newcomers unfolds across eight phases/domains: (a) condition in homeland; (b) migration context; (c) new environment; (d) challenging events; (e) adaptation responses; (f) return migration; (g) short-term outcomes (e.g. sociocultural integration or assimilation into the host society); and (h) long-term outcomes (e.g. economic or educational advancement or family or personal well-being).

The salience of stress and coping is particularly highlighted within the domains of ‘challenging events’ (phase d) and ‘adaptation responses’ (phase e). Under ‘challenging events’, Castro and Murray (2010) observed and contended that key stressors faced by immigrants can arise from having to face racial/ethnic discrimination, novel opportunities, illness, growth milestone, and unexpected or uncontrollable happenstance. These authors asserted that the extent to which immigrants appraise these events, in terms of opportunity vs. threat, would ultimately determine their well-being (i.e. distress, mental disorders, and health problems). In view of these stressors, resilient coping behaviors are manifested under the next phase, the ‘adaptation responses’ domain, when coping attempts are activated to deal with the emerging stressors. While in Berry's (1997) acculturation theory coping behaviors are defined in specific terms (i.e. problem-focused, emotion-focused and avoidance coping) as reviewed in the last section, Castro and Murray (2010) conceptualize resilience coping more broadly in their stress–appraisal–coping model. That is, resilience coping behaviors pertain to immigrants' personal competence or skills that can enable them to attain desirable goals and favorable short- and long-term adaptation outcomes (phase g and h). These can include immigrants' ability to effectively engage in decision-making, self-control and self-regulation.

#### Stress and coping grounded theory for recent immigrants by Yakushko (2010)

While the foregoing theoretical propositions on coping and cultural adaptation are predominated by conceptual analysis and synthesis of the literature and, in some cases, field or clinical observations, the final model proposed by Yakushko (2010) reviewed was directly derived from an empirical qualitative study of immigrant participants. Specifically, this Stress and Coping theory was developed based on a grounded theory approach by interviewing immigrant leaders in a Midwestern city of the USA. From this standpoint, this ‘bottom-up’ and data-driven approach, going from data to findings to a theoretical framework, makes this Stress and Coping framework particularly unique. As contended by the author the primary intent of the research was to generate ‘an integrated and empirically derived model that seeks to bring together information obtained directly from the immigrants and its leaders’ (p. 257; Yakushko, 2010) and to address how immigrants respond to diverse stressors during their adjustment in the new host cultural environment.

The author and her research team interviewed 20 representatives from various ethnic immigrant communities which included Latin American, Middle Eastern, African, Asian, and Eastern European groups residing in Lincoln, Nebraska. On the basis of their qualitative data analysis, the author proposed a process model of coping and acculturation/cultural adaptation that comprises of five domains: (a) causal conditions (reasons, status, and expectations associated with migration); (b) central phenomenon (sources and types of stress); (c) coping patterns (constructive vs. detrimental coping methods); (d) context (individual to family to community context); (e) intervening conditions (available resources and context-specific values); and (f) adaption consequences (cultural, family, social, legal, health, work/educational, or empowerment outcome) (Yakushko, 2010).

Within this framework, acculturation-related stresses are identified in the second step of the cultural adaptation process, namely the ‘central phenomenon’. This process is followed by immigrants' coping efforts to respond and manage these stressful experiences as indicated in the third step of the process under ‘coping patterns’. Yakushko's (2010) findings pointed to broad categories of coping behaviors used by the immigrant participants, which included the use of ‘individual values’, ‘connections’, ‘giving’, ‘personal development’, ‘distractions’, and ‘help-seeking’ by immigrants. This theory hypothesizes that immigrants' selective coping strategies act as the critical ‘mediators’ between stress (in the ‘central phenomenon’ step) and the adaptation outcomes (in the final ‘consequences’ step). Importantly, the author noted that the quality of the coping methods used by immigrants can be either constructive/adaptive or detrimental/maladaptive. Furthermore, a given coping strategy, such as coping through religion and spirituality, can have either constructive (e.g. being used as a motivating method for change) or detrimental (e.g. being used as an avoidance) effects on immigrants depending on how it is being applied in the face of the stressor. Given that Yakushko's (2010) stress and coping grounded theory was generated based on a single qualitative study with migrant participants in a very specific geographical area in the USA and that it is a relatively new conceptual scheme, the broader relevance and applicability of the theory to coping and cultural adaptation of migrants elsewhere awaits further empirical verification. Clearly, more research on this theoretical model is needed.

### Summary and evaluation of the theoretical and conceptual models

As a whole, all four models are credited for assuming a comprehensive, ‘ecological’ perspective in accounting for the interactions between characteristics within the acculturating individual's ‘personal system’ and external forces in the person's environment when considering coping and cultural adaptation. Berry's model is particularly helpful in highlighting the attitude of the host society in affecting the acculturating individual's stress experiences and the acculturation strategy the person is likely to adopt in response. The model also goes further by identifying and characterizing the phenomenon of acculturative stress – a common source of stress that demands and evokes migrants' coping responses. On the other hand, Yakushko's (2010) model is valuable in identifying and categorizing diverse range of coping behaviors often used by immigrants in responding to cultural changes. The author does so by describing these coping methods in explicit terms. This type of information is especially useful for coping researchers when delineating and assessing migrants' coping repertoires (Kuo, Roysircar, & Newby-Clark, 2006).

Castro and Murray's (2010) theory is enlightening in that it introduces ‘resilience’, a characterlogical attribute, to help understand and explain why some migrants enjoy faster and more successful cultural adaptation than others. They do so by framing the cultural adaptation process from a developmental perspective. At the same time, this theory also leaves unanswered questions about the role and the extent of personal predispositions (i.e. resilience trait) play in predicting the quality of acculturation/cultural adaptation among different migrants. Finally, while Cervantes and Castro's (1985) model stands as a pioneering attempt to discern the stress–coping–mental health association for immigrants, the model was ethnospecific for Mexican Americans and it was grounded in earlier literature in this area. Thus, the model's applicability to more recent Mexican Americans and other non-Mexican American migrants is not clear at this point.

That being said, the foregoing review of the four theoretical models is nonetheless a valuable starting point for researchers to move towards a greater integration of theory, research, and practice that concern with coping, acculturation, and adaptation for migrants. In particular, these models lay out broad structural and conceptual ‘roadmaps’ to aid researchers and practitioners in better understanding otherwise highly complex and intricate interactions among these and other adjustment- and adaptation-related variables.

### Empirical research on coping and acculturation

Theoretical models like the ones reviewed above offer predictions in the relationship among stress, coping, acculturation, and psychological adaptation for migrating individuals. According to Berry's (1997) acculturation strategies framework, for instance, an individual undergoing cultural transition is likely to experience significant changes in language, behaviors, cognitions, personality, identity, attitudes, psychological well-being; he or she is also regularly confronted with stress that demands coping to respond to the flux of changes (Berry, 1997; Redfield et al., 1936; Zheng & Berry, 1991). On the basis of this assertion, other scholars have hypothesized that: (a) migrants' preferred patterns of coping behaviors used to manage and counter their stresses, either general or adjustment-related stresses, should vary depending on migrants' extent of acculturation levels and the acculturation strategies they adopt; and (b) these preferred coping strategies would have varying impacts on migrants' psychological and mental health well-being (Kuo et al., 2006; Ward, 2001).

Grounded in these observations with the intent to bridge the current theories to empirical research on coping, acculturation, and psychological adaptation, this paper now turns to examine relevant empirical studies on coping and acculturation among various migrant populations. Specifically, the ensuring section of this review narrows its focus on published studies in English that had: (a) investigated the characteristics of coping behaviors of various acculturating groups; (b) examined and implicated the role of coping in relation to acculturation for the immigrant group or groups under study; and (c) assessed the impacts of coping on acculturation of migrants in terms of psychological, mental health, and related outcomes.

To identify the most relevant published studies for this review, a thorough search was conducted on the most comprehensive psychological bibliographic database, *PsychInfo*. Multiple combinations of keywords, including ‘acculturation’, ‘cultural adaptation’, ‘cultural adjustment’, ‘coping’, and ‘stress response’, were entered in the database for the search. Further, published articles familiar to the author of this article and had met the inclusion criteria stated above were additionally included in the review. These efforts yielded a limited number of published articles on the topic. For heuristic purposes, they are organized and presented in two categories: (a) studies that compared cultural group differences in coping patterns and preferences; and (b) studies that examined coping's relationship to measured or inferred acculturation levels/statues of migrant groups.

#### Differences in coping patterns and preferences among acculturating migrant groups

The earliest empirical evidence pointing to the association between preferred patterns of coping and acculturation stems from between-cultural group comparative studies on coping behaviors. As a case in point, Mena, Padilla, and Maldonado (1987) examined the coping patterns among immigrant college students of four different generation statuses in the USA. The results showed that the generation status of immigrant students had an effect on their experiences with acculturation stress as well as on the kinds of coping strategies they preferred. The late immigrant group reported a greater use of active coping methods than individuals from early immigrant and later-generation backgrounds. In contrast, the second- and the third-generation immigrant participants reported to utilize more social network as a coping method as compared to the first- and the mixed-generation groups. The authors surmised that being more acculturated in the US second- and third-generation immigrants would likely have more interpersonal and social resources at their disposal for coping with stress. Similarly, in a Canadian study of cultural adjustment among individuals of varying immigration statuses, Zheng and Berry (1991) found that Chinese sojourners, being the most recent and the least acculturated newcomers to Canada, reported more areas of stresses and problems (e.g. homesickness, loneliness, etc.) than Chinese Canadian students, and European Canadian students. The same group also used more positive coping (e.g. more tension reduction and information-seeking), and less passive coping (e.g. wishful thinking and self-blame) than European Canadian students.

The nature of stress and coping has also been found to be related to acculturation among different groups of student population distinguished by their immigration statuses. For instance, Chataway and Berry (1989) investigated the relationship between coping and cultural adaptation among university students of Hong Kong Chinese, French Canadian, and English Canadian backgrounds. In this study, it was shown that as compared to French and English Canadian students, Hong Kong Chinese students being less acculturated than the other two groups reported greater acculturative stress (e.g. anxiety, prejudice, adjustment difficulties, and social support) as predicted. This latter group also used coping strategies differed from Canadian students and were more likely to engage in less positive thinking and less tension reduction coping methods in dealing with their stress. The same group further demonstrated poorer adaptation outcomes as compared to the French and English Canadians. That is, Chinese students showed poorer health and lower satisfaction with their coping abilities. Collectively, these three studies above suggest that cultural group differences in coping patterns can be affected by migrants' generation status and their immigrant status, both of which are proxy variables for acculturation.

Other coping and acculturation studies have focused on differential coping repertoires and their effects on adjustment among migrants of differing acculturation levels within the same ethnic group. In a study by Noh and Kaspar (2003), the authors investigated the role of coping, acculturation and ethnic support in moderating the relationship between acculturative stress (i.e. perceived discrimination) and depression in a sample of Korean immigrants in Toronto, Canada. When the effect of coping in responding to perceived discrimination on depression was examined, among more acculturated Korean immigrants, the use of problem-focused coping strategies were found to be helpful in buffering the negative impacts of discrimination on these individuals (Noh & Kaspar, 2003). However, for less acculturated Korean immigrants, the same coping methods did not produce the same effect. The authors stipulated that coping preference and effectiveness vary for immigrants with different degrees of acculturation and social resources. In particular, it was reasoned that with increased levels of acculturation, Korean immigrants' choice of coping responses to discrimination might have approximated to the normative coping pattern (i.e. problem-focused coping) of the more individualistic host Canadian society.

In a unique qualitative study, Yoshihama (2002) examined coping responses to domestic violence faced by Japanese women of different acculturation backgrounds living in the USA. The author found that the US-born Japanese battered women utilized more active means of coping methods (e.g. confronting the perpetrator or seeking help from friends) and considered active coping more effective than their less acculturated, Japanese-born battered counterparts in dealing with domestic violence. In contrast, the Japanese-born group adopted more passive coping strategies, including minimizing one's problem or focusing their attention on the positives of the abuser, than their more acculturated US-born counterparts. Interestingly, while US-born women's perceived effectiveness in their active coping was also linked to lessened psychological distress, the opposite effect was true for Japanese-born battered women. The latter group found the same active coping approach to be linked to more psychological distress for them. In fact, Japanese-born women's perceived effectiveness in passive coping was linked to lessened psychological distress, but the same approach had no effect for US-born Japanese women. The author hypothesized that for the Japanese-born battered women active, problem-oriented coping is intrinsically not compatible with traditional Japanese cultural values, even in the case of dealing with partner violence. The study points to the effect of within-group variability in coping preferences as a function of immigrants' nativity status (native vs. foreign-born) and the implied differences in the socialization and acculturation among these immigrant women.

Together Noh and Kaspar's (2003) and Yoshihama's (2002) studies suggest that not only can migrants with varying acculturation experiences differ in their typical patterns of stress responses and coping, but also the functional utility of various coping strategies can have divergent effects on migrants depending on their extent of acculturation. Conceptually, these results lend valuable support to Yakushko's (2010) Stress and Coping Grounded Theory discussed earlier. The theory stipulates that the coping patterns (domain c) of immigrants with different migration and acculturation statuses would be affected by specific contexts (domain d; e.g. ethnic and cultural contexts) and intervening conditions (domain e; values and resources) associated with their specific acculturation backgrounds. In another word, migrants with different degrees of cultural experiences in the host culture (e.g. native vs. foreign-born immigrant) are expected to cope with a similar stress differently because they are shaped by divergent cultural forces, expectations, values and available resources. Thus, these findings are encouraging from the empirical as well as the theoretical standpoint as they offer some initial evidence in support of a social, cultural, and contextual model of coping (Aldwin, 2007; Folkman & Moskowitz, 2004; Heppner, 2008) and are consistent with the conclusions of recent reviews on cross-cultural and cultural coping literature (Kuo, 2011, 2013). However, the number of existing empirical studies on this subject matter is still quite limited and the findings should be viewed as preliminary and taken with caution.

#### Coping variations as a function of varying degrees of acculturation among migrants

Another collection of empirical studies linking coping to acculturation is found in research examining coping of immigrants and members of ethnic groups in relation to the measured acculturation levels of these individuals. Given that migrants of different generation and immigration statuses (i.e. proxy indication of acculturation) appear to exhibit different coping behaviors as suggested by the studies reviewed in the last section, it stands to reason that migrants' actual acculturation levels can and should impact on *how* and *what* they prefer to use to cope with general as well as adaptation-related stresses. It can be hypothesized that preferred or normative coping behaviors among immigrants and ethnic groups can vary along the dimension of their acculturation level. A handful of empirical research has indeed attempted to explore this relationship between coping and acculturation in different migrant and ethnic samples.

Yoo and Lee (2005) studied the role of ethnic identity, a closely related construct to acculturation, and the use of approach-type coping among Asian American college students in the context of dealing with perceived racial discrimination. These author defined and measured approach-type coping based on coping strategies that rely on social support, cognitive restructuring, and problem-solving. Overall Asian Americans with a strong ethnic identity were found to be more likely to use social support and problem-solving coping in dealing with experience of discrimination than those who reported a weak ethnic identity. However, cognitive restructuring or problem-solving coping had a positive buffering effect from stress only for participants with a high level of ethnic identity and those who perceived discrimination to be infrequent and of low intensity.

One specific study by Kuo and his colleagues actually measured acculturation along with a culture-based measure of coping in a sample comprised of three cohorts of Chinese adolescents in Canada (Kuo et al., 2006). The sample included: Chinese Canadians, Late-Entry Chinese Immigrants, and Chinese Sojourners. These researchers postulated that the relationship between generational/immigrant status and preferred coping approaches might actually be mediated by these adolescents' degrees of acculturation. As predicted, significant cohort effects and differences in coping and acculturation were identified. Less acculturated cohorts (e.g. Chinese Sojourners) preferred more collective coping and avoidance coping methods in managing their acculturative stresses than did those in more acculturated cohorts (e.g. Chinese Canadians). It was interpreted that less-acculturated adolescents adhered more strongly to traditional Asian values of collectivism and interpersonal harmony, which in turn prompted a greater utilization of collective and avoidance (e.g. not rocking the boat) coping. Together the findings of Yoo and Lee's (2005) and Kuo et al.’s (2006) studies suggest that coping preference and efficacy for ethnic and migrant youth do vary along the dimension of acculturation and the social resources available to them in the dominant/host society. Once again, these results are in-line with the prediction by Yakushko's (2010) Stress and Coping Grounded Theory. They point to the existence of divergent preferred coping repertoires among immigrants of varying degrees of acculturation (Kuo, 2011), owing to different cultural and contextual influences and resources which give rise to their coping decisions (Aldwin, 2007; Yakushko, 2010).

The cultural adaptation process and responses to acculturative stress experienced by young international students, known as ‘unaccompanied sojourners’, was examined in a model testing study by Kuo and Roysircar (2006). With a sample of 201 adolescent sojourners from Taiwan studying in Canada, the study tested several competing models with the structural equation modeling procedure. The models tested these sojourners' perceived prejudice (or discrimination), education-related acculturation, and age of arrival in the host country as the antecedent predictors of their acculturative stress and ethnic identity. The results showed that while sojourners' perceived prejudice positively predicted acculturative stress, education-related acculturation negatively predicted their acculturative stress. Of particular relevance to coping and acculturation, the model revealed that sojourners' ‘interpersonal competence’, as measured by the participants' sense of social support and interpersonal effectiveness, acted as a significant mediator in the link between perceived prejudice and acculturative stress and between education-related acculturation and acculturative stress.

These observations by Kuo and Roysicar (2006) find support in a study of acculturative stress among first-generation adult Hispanic immigrants (*N* = 96) in the USA by Torres and Rollock (2004). The study tested demographic variables, acculturation, coping, and intercultural competence of Hispanic adults as predictors of acculturative stress among this migrant group. The authors defined and measured coping in terms of general coping, active coping, emotional maintenance, and autonomy from others. Intercultural competence, on the other hand, was assessed in terms of the participants' social, cultural, academic, and career competence in the US host culture. In another word, it is a form of interpersonal competency on the part of migrants specifically pertaining to competencies within the context of cultural interactions as described by Kuo and Roysircar (2006). The study found that both intercultural competency and general coping were significant negative predictors of acculturative stress for these Hispanic migrants after accounting for the effects of various demographic and acculturation-related variables. Conceptualizing from a stress and coping perspective, these findings suggest that adaptation and coping strategies through interpersonally as well as interculturally competent behavioral and cognitive responses can serve to buffer sojourners and immigrants against acculturative stress. These interpersonally and cross-culturally competent coping skills encompass migrants' ability to effectively mobilize their available social support and resources, to actively engage in dealing with their loneliness and isolation, and to productively negotiate their way through cultural differences and conflicts with the host culture.

Other scholars have considered different modes of ‘acculturation strategies’ adopted by migrants, namely *assimilation*,* separation*,* marginalization*, and *integration*, to be in and of themselves means of coping for adjusting to a new cultural context (Berry, 1997; Ward, 2001). In a recent study, Yoon et al. (2012) attempted to address the question on the extent to which acculturation and enculturation (i.e. cultural learning of one's heritage background) affect adaptation consequences of racially and ethnically diverse populations based on mental health indicators (e.g. depression, anxiety, psychological distress, negative affect, and positive affect). Based on a meta-analysis of 325 studies identified in the literature by the authors, coping directly associated with these different modes of acculturation strategies was shown to be connected to differential mental health outcomes for migrant and ethnic populations. The results conclude that acculturation is linked to reduced negative mental health and increased positive mental health, but enculturation is linked to increased positive mental health only. In terms of specific acculturation strategies, consistent with Berry's (1997) theoretical prediction, integration or biculturalism was found to be the most advantageous or ‘healthiest’ acculturation orientation in relation to mental health for culturally diverse racial/ethnic minorities and immigrants. Incidentally, this meta-analytical study revealed that the combination of engaging in external acculturation (e.g. adopting language or behavior of the host culture) and internal enculturation (e.g. maintaining one's racial and ethnic identity) is the most advantageous coping approach for migrants and members of ethnic groups in response to cultural changes in terms of psychological well-being.

Together these foregoing empirical studies on coping and acculturation do substantiate a robust link between these two constructs for migrants and ethnic populations as postulated by the theoretical models reviewed previously. Broadly speaking, these data and findings have offered further empirical evidence in support of the stress, coping, and adaption theoretical perspective on acculturation (Berry, 1997; Ward, 2001) and solidified the critical role coping plays in the overall process of intercultural contact and acculturation for migrants.

## Discussion

This article is intended to be among the first scholarly attempt to examine current, cumulative psychological theories and research on coping, acculturation and psychological and mental health adaptation among migrants. As such, the present review contributes to the existing literature by providing an initial effort to compile and summarize relevant conceptual and empirical works on this increasingly important subject of coping and acculturation. A number of key implications are identified in this current review; these insights and observations have direct or indirect implications on future coping-acculturation research on migrants and members of ethnic minority.

First, in terms of theoretical implications, four conceptual models of stress–coping–acculturation–adaptation were described and reviewed in this paper. Albeit limited in number the advent and the articulation of these acculturation and cultural adaptation theories are indicative of the increasing relevance and maturity of this area of research. Collectively, these theoretical models provide the crucial conceptual ‘roadmap’ or ‘building blocks’ upon which future research on coping behaviors, acculturation, and adaptation can be built and advanced. As shown in this review, despite their differences in specific content, configuration, and sequential relationships among key variables, all four models share in common the emphasis on coping as: (a) a principle determinant of cultural adaptation and acculturation for migrants; (b) a critical mediator or moderator between stressors (general or acculturative) faced by migrants and their psychosocial adaptation in the host society; and (c) a process that is profoundly shaped and influenced by migrants' unique culture characteristics and social contexts (Aldwin, 2007; Castro & Murray, 2010; Kuo, 2011). In this regard, these prevailing stress–coping acculturation/cultural adaptation theoretical frameworks are conceptually aligned with the larger stress-coping psychological theory as originally proposed by Lazarus and Folkman (1984).

Second, these emerged theoretical models have laid out much-needed directions and conceptual schemes to help interpret and assimilate the findings of past and present empirical research and to help guide future studies in this area. These structured models can help inform researchers to make more rational interpretations of their research findings related to coping and cultural adaptation among migrants post hoc after a research is completed. These models can further afford researchers more accurate predictions when they discern and study the coping-acculturation processes and pathways prior to conducting a research. For instance, these theories can guide researchers to formulate new and germane research questions concerning coping-acculturation-adaptation and/or to identify potential, relevant factors in this pathway when planning for a study. On this point, in fact Castro and Murray (2010) observed a lack of theory-driven empirical examination and research of what and how migrant individuals undergoing cultural changes and cope in relation to their acculturation experiences; thus, they strongly advocated more scholarly efforts in this direction.

Third, the review and the analysis of existing empirically based studies on coping and acculturation/cultural adaptation in this article point to a number of intriguing facts and findings. As expected, overall the studies reviewed in this present article point to a robust yet complex relationship between coping and cultural changes for migrants. In sum, the findings from the studies surveyed highlight that: (a) coping plays an integral role in the process of acculturation, particularly in mitigating the effects of both acculturative stress and non-acculturative stress for migrants; (b) while the use of active and problem-focused coping strategies by migrants promotes positive adaptation and emotional well-being, the use of avoidance coping strategies hinders adaptation and can lead to negative effects on adjustment outcomes; and (c) varying degree of acculturation is related to differentially preferred patterns of coping behaviors among immigrant and ethnic minority individuals and groups (Kuo et al., 2006; Noh & Kaspar, 2003; Zheng & Berry, 1991). In this respect, the cumulative research findings have lent some initial empirical support to the stress-based theoretical models of acculturation discussed earlier in this paper. For instance, both the Resilience-based stress–appraisal–coping model by Castro and Murray (2010) and the Stress and Coping Grounded Theory for Recent Immigrants by Yakushko (2010) stipulate that migrants' cognitive appraisal and coping behaviors in responses to stresses and adversities during the cultural adaptation process can be either constructive/adaptive or detrimental/maladaptive in nature. Both theories contend that the quality of migrants' perception of their stress and their cumulative coping efforts over time are considered to be important in predicting their eventual adaptation experiences in the host culture.

### Limitations of the existing literature and implications for future research

The findings and insights generated in the current paper, however, need to be viewed with caution, given a number of limitations associated with the prevailing research on coping, acculturation, and psychological and mental health adaptation. First of all, it is noted that while the theories reviewed in this paper hypothesize stress, coping, acculturation and adaptation to be a temporal and longitudinal process (see Berry, 1997 as an example), empirical studies on coping and acculturation continue to base heavily on cross-sectional survey design. This theory-methodology mismatch limits the potential of fully capturing and comprehending the multifaceted, sequential and progressive nature of stress–coping–adaptation experienced by migrants. Therefore, methodologically speaking, longitudinal studies measuring and tracking how migrants' coping behaviors and cultural adaptation evolve across different phases of their acculturation process are highly desirable. This type of long-term investigation should be a focus of coping and acculturation research in the future.

Secondly, even though the emerging empirical findings on coping and acculturation among migrants are informative and encouraging, it is nevertheless important to acknowledge that cumulative results are still quite preliminary at this point. Only a few empirical studies identified in this review actually measured and examined coping, acculturation and adaptation and the relationships among them directly and simultaneously in the study (see Noh & Kaspar, 2003 as an example). A lack of simultaneous examination of critical variables germane to the coping-acculturation-adaptation link in empirical research relegates the interpretation of a study's results to post hoc inference and guesswork, and thus restricts the confidence one can place in the results. It is critical then for future coping-acculturation research to clearly define, measure, and hypothesize the antecedents, the mediators/moderators, and outcome variables in this stress–coping–acculturation–adaptation causal link in studying the experiences of migrants (Kuo, 2011). For instance, in Noh and Kaspar's (2003) study of coping with depression among Korean immigrants in Toronto, the authors measured perceived discrimination (antecedent stressor), emotional coping (coping moderator), acculturation and ethnic social support (acculturation moderator), and depression (mental health outcome) in the same study. Additionally, future research on migrants' coping and acculturation should also consider examining other stress-related extraneous variables of acculturating migrants, such as social support, family support, structural resources (e.g. community or mental health services). Once again, the theories reviewed in the previous section can serve as conceptual guides to inform researchers on *what* critical variables and *how* to include them in a given study.

Thirdly, the current review of prevailing empirical studies on coping and acculturation reveals that the existing corpus of research in this area appears to be quite piecemealed. That is, there is a lack of systematic and programmatic effort to study coping and cultural adaptation among acculturating groups. As a result, there is currently no clear sequential or continuous research attempts to investigate coping and acculturation among migrants over multiple studies. This lack of continuity in this area of research may be partly attributable to the fact that many of the published studies on coping among migrants reviewed in the current article were not grounded in or driven by clear theoretical rationales. Thus to forge a sustainable programmatic research on coping, acculturation, and adaptation this current body of literature would greatly benefit from moving toward a theory-driven approach in developing empirical research. Researchers should consider applying and validating the theoretical models reviewed in this article in future study. These theories can serve as the conceptual basis for researchers to come up with specific research questions, to design the study, to formulate appropriate methodology, and to even verify and/or modify the original theory.

Finally, as indicated in this review article, the existing literature on coping and acculturation is heavily dominated by empirical investigations of the experiences of migrants in North America (i.e. USA and Canada). As well, the prevailing studies have focused mainly on the experiences of voluntary migrants (i.e. immigrants, international students/sojourners, and members of ethnic minorities). None of the studies reviewed included specific involuntary migrants, such as refugees. Furthermore, all of the research reviewed in this article is based on the literature of international migration. To what extent does the relationship between coping and acculturation for international migrants apply to internal migrants cannot be ascertained at this point. These issues clearly represent gaps in the existing literature on coping and acculturation and they call for additional research attention.

## Conclusion

Considering the increasing prevalence of mass migration and movement of people across regional, national and continental boundaries as globalization continues, the need to better understand how migrants engage in cross-cultural changes, how they respond to and cope with stresses and challenges that come with these acculturation processes, and how their coping and adaptation strategies are impacting their health and well-being is more pressing than ever. The answers to these questions have far-reaching implications for practice, research, and policy implementation for academics, community agencies, and governments who are concerned with the welfare of migrants worldwide. This present theoretical and empirical review is a deliberate attempt towards addressing these questions. It is hoped that this review and synthesis will engender further dialogues, discussions and debates about the stress–coping approach to acculturation research and serve as a basis to stimulate further research to understand coping and its relationship to cultural adaptation process and outcome for migrants.
